# Biochemical Compounds, Antioxidant Capacity, Leaf Color Profile and Yield of Basil *(Ocimum* sp.) Microgreens in Floating System

**DOI:** 10.3390/plants12142652

**Published:** 2023-07-14

**Authors:** Mohammad Reza Fayezizadeh, Naser Alemzadeh Ansari, Mohammad Mahmoudi Sourestani, Mirza Hasanuzzaman

**Affiliations:** 1 Department of Horticultural Science, Faculty of Agriculture, Shahid Chamran University of Ahvaz, Ahvaz 61357-43311, Iran; 2 Department of Agronomy, Faculty of Agriculture, Sher-e-Bangla Agricultural University, Sher-e-Bangla Nagar, Dhaka 1207, Bangladesh; 3 Kyung Hee University, 26 Kyungheedae-ro, Dongdaemun-gu, Seoul 02447, Republic of Korea

**Keywords:** anthocyanin, carotenoids, chlorophyll, chroma, flavonoid, healthy food, phytochemical, polyphenols, vitamin C

## Abstract

Basil is a great source of phytochemicals such as polyphenols, vitamin C, anthocyanin, and flavonoids. In this work, the biochemical compounds, antioxidant capacity, leaf color profile, and yield of 21 cultivars and genotypes of basil microgreen were investigated. Results showed that the highest antioxidant potential composite index (APCI) was measured in Persian Ablagh genotype (70.30). Twenty-one basil genotypes were classified into four clusters, including cluster 1 (lowest antioxidant capacity and total phenolic compounds), cluster 2 (lowest anthocyanin, vitamin C and APCI index), cluster 3 (highest vitamin C, total phenolic compounds, antioxidant capacity and APCI index), and cluster 4 (highest levels of anthocyanin). The principal components analysis (PCA) of basil genotypes showed diversity in terms of phytochemical components, and F1, F2, F3, and F4 explained the variation at the rate of 78.12%. The average annual temperature of the origin of basil seeds plays an important role in the synthesis of antioxidant content. Most of the seeds with moderate origin had a higher APCI index. The Persian Ablagh genotype, Violeto, and Kapoor cultivars can be recommended, according to their APCI index and yield. These cultivars can be used individually or in different ratios to produce different biochemical substances with different concentrations for various purposes.

## 1. Introduction

Basil (*Ocimum* sp), which originates from India, Africa, and Southern Asia, among the different botanical families, is one of the most valuable aromatic and medicinal plants (AMPs) and has about 60 varieties, which are identified and classified in the genus Ocimum in the plant family Lamiaceae [1]. Basil is a rich source of phytonutrients, including vitamin A, vitamin C, calcium, magnesium, potassium, zinc, and iron, and, due to the enriched presence of secondary metabolites such as essential oils, tannins, phenols, flavonoids, anthocyanins, carotenoids, and steroids, it has long been recognized for its medicinal and health properties [2]. Scientific studies provide evidence of the anti-inflammatory properties, antioxidant activity, pain-reducing effect, fever-reducing effect, liver-protecting effect, anti-cancer activity, diabetes-preventing effect, blood-vessel-protecting effect, stress-reducing effect, and immune-boosting properties of basil [3]. As a result, growing basil as a microgreen can be a good option, considering its many properties, but considering the diversity, choosing cultivars and genotypes of this plant requires more research.

In the recent research conducted on the basil family, it was determined that the basil cultivars were different in terms of photosynthetic pigments, and that the purple basil cultivar contained 166% and 67% higher total phenols and flavonoids than the green basil variety, respectively [4]. Moreover, Incrocci et al. [5] reported that basil cultivars (*Ocimum basilicum* L. cv. Tigullio and cv. Red Rubin) had no significant difference in total chlorophyll and carotenoids, but Red Rubin basil had a higher content of nitrates and total phenols. In another study, the biochemical compounds and nutritional elements of green and purple basil microgreens were evaluated, and the results showed that the cultivars had a significant difference in yield and leaf nitrate content, and that purple basil had a higher yield (45% more) of nitrates and total phenols than the green basil [6]. In a study of the growth and development of 28 different types of microgreens, it was shown that basil microgreens, especially lemon basil (*Ocimum* × *Africanum*) and dark purple basil (*Ocimum basilicum* Cv. Dark Opal), as well as purple mint (*Perilla frutescens*), have lower growth and yield compared to other cultivars. Cultivars of the Lamiaceae family, which include green basil, purple basil, lemon basil, and perilla, did not exhibit significant differences in terms of dry matter, but the yield of perilla was higher than those of other basil cultivars [7]. In the past few years, much research has been done on different varieties of mature basil, but there is little information about the microgreen stage, so the response of different cultivars in terms of performance, biochemical properties, and antioxidants in the cultivation of microgreens basil needs more research.

The production of microgreens has been introduced in the 21st century due to the short harvest time, high concentration of vitamins, nutrients, and secondary metabolites, and high commercial value. Due to their high content of phytochemicals and antioxidant compounds such as carotenoids, ascorbic acid (vitamin C), phylloquinone (vitamin K1), γ-tocopherol, total phenolic compounds (TPC), antioxidants, and macro- and micronutrients, they were called “Superfood” [8,9,10]. 

Microgreens are especially important, usually consumed in the spring when fresh fruits, vegetables, and herbs are less available. Studies on the relationships between the genotypes and the secondary metabolites of basil microgreens are limited, but these products are commonly offered for sale. This study was carried out to expand the knowledge about antioxidant capacity, plant phytochemicals, and leaf color profile. Such information is important for consumers in making purchases or for health service providers in conducting research or providing advice about dietary supplements. In this study, we sought to determine the effects of seed origins on the biochemical content and antioxidant compounds of basil microgreens, so that we can determine the diversity among microgreens. Therefore, the object of this study was to determine the best cultivars and genotypes of microgreen basil based on yield, biochemical and antioxidant compounds, including vitamin C, polyphenols, flavonoids, carotenoids, antioxidant capacity, anthocyanin, and nitrate, and leaf color profile. Some of the microgreen basil genotypes selected in this experiment were investigated for the first time.

## 2. Results

### 2.1. Chlorophyll and Carotenoid Content

As shown in Table 1, the Kapoor cultivar had the highest amount of chlorophyll a (0.93 mg g^−1^ FW), chlorophyll b (0.24 mg g^−1^ FW), and total chlorophyll (1.18 mg g^−1^ FW). The Amethyst cultivar had the lowest amount of chlorophyll a (0.27 mg g^−1^ FW), chlorophyll b (0.08 mg g^−1^ FW), and total chlorophyll (0.35 mg g^−1^ FW). The highest and lowest carotenoid contents were measured in the Hoary (0.33 mg g^−1^ FW) and Dark Opal (0.06 mg g^−1^ weight) cultivars, respectively. The total chlorophyll content had a positive and significant correlation with antioxidant capacity (r = 0.544 *), TPC (r = 0.481 *), nitrate content (r = 0.500 *), and APCI index (r = 0.543 *). The content of carotenoids had a positive and significant correlation with the APCI index (r = 0.57 9 **) (Table 2). 

### 2.2. Vitamin C

According to Table 1, the highest and lowest contents of vitamin C were obtained in Kapoor (2.40 mg AA g^−1^ FW) and lemon basil (1.03 mg AA g^−1^ FW), respectively. Vitamin C had a positive and significant correlation with the APCI index (r = 0.459 *) (Table 2).

### 2.3. Antioxidant Capacity (AC)

The highest and lowest antioxidant capacities were measured in the Mobarake genotype (86.76% DPPH inhibition) and Amethyst cultivar (8.26% DPPH inhibition), respectively (Table 1). Antioxidant capacity had a positive and significant correlation with polyphenol compounds (r = 0.669 **), nitrate content (r = 0.434 *), and APCI index (r = 0.667 *) (Table 2).

### 2.4. Total Phenolic Compounds (TPC)

The highest and lowest TPCs were obtained in the Ablagh basil genotype (1463.79 mg GA 100 g^−1^ FW) and the thyrsiflora cultivar (135.88 mg GA 100 g^−1^ FW) (Table 1). Polyphenol compounds had a positive and significant correlation with the APCI index (r = 0.561 *) and yield (r = 0.448 *) (Table 2).

### 2.5. Total Flavonoid Contents (TFC)

The highest and lowest contents of flavonoids were obtained in the Amethyst cultivar (7.95 mg CAE 100 g^−1^ FW) and Basilico Rosso cultivar (0.63 mg CAE 100 g^−1^ FW), respectively (Table 1). Flavonoid content was positively correlated with nitrate content (r = 0.495 *) (Table 2).

### 2.6. Anthocyanin

The highest and lowest anthocyanin contents were obtained in the Ablagh basil genotype (26.19 mg 100 g−1 FW) and Kapoor cultivar (12.83 mg 100 g−1 FW), respectively (Table 1). Anthocyanin had a positive and significant correlation with a* (r = 0.472 *) and APCI index (r = 0.452 *) and had a negative and significant correlation with b* (r = 0.546 *) and Chroma (r = 0.543 *) (Table 2).

### 2.7. Nitrate

The highest and the lowest amounts of leaf nitrate were obtained from the lettuce leaf basil (866.54 mg kg^−1^ FW) and Persian green basil genotypes (95.62 mg kg^−1^ FW), respectively (Table 1).

Based on the results of the average comparison in Table 3, microgreen basil cultivars and genotypes had significant differences in terms of variables determining color intensity. 

**Table 2:** Correlation coefficient* between studied characters.

9	Chla	Chlb	Chla + b	Car	Vit C	AC	TPC	TFC	ACNs	Nit	a*	L*	b*	Hue	Ch	AI	Y
Chla	1																
Chlb	0.987 **	1															
Chla + b	0.999 **	0.992 **	1														
Car	0.308	0.350	0.326	1													
Vit C	0.250	0.292	0.260	0.215	1												
AC	0.547 *	0.544 *	0.544 *	0.161	0.385	1											
TPC	0.476 *	0.466 *	0.481 *	0.113	0.186	0.669 **	1										
TFC	−0.064	−0.096	−0.071	0.137	−0.120	0.066	−0.210	1									
ACNs	0.084	0.047	0.080	0.060	−0.040	0.130	0.295	0.199	1								
Nit	0.497 *	0.494 *	0.500 *	0.221	−0.066	0.434 *	0.367	0.495 *	−0.059	1							
a*	−0.123	−0.180	−0.140	−0.163	0.019	−0.129	0.234	0.250	0.472 *	−0.116	1						
L*	0.119	0.157	0.126	−0.050	−0.171	0.083	−0.203	−0.367	−0.372	−0.027	−0.814 **	1					
b*	0.163	0.212	0.174	0.039	−0.133	0.056	−0.210	−0.187	−0.546 *	0.168	−0.884 **	0.924 **	1				
Hue	−0.189	−0.219	−0.201	−0.243	−0.014	−0.187	0.288	0.101	0.309	−0.135	0.910 **	−0.666 **	−0.716 **	1			
Ch	0.155	0.209	0.168	0.082	−0.090	0.047	−0.190	−0.224	−0.543 *	0.155	−0.927 **	0.908 **	0.990 **	−0.762 **	1		
AI	0.536 *	0.537 *	0.543 *	0.579 **	0.459 *	0.667 **	0.561 **	0.379	0.452 *	0.529 *	0.181	−0.320	−0.259	0.055	−0.242	1	
Y	0.301	0.302	0.302	0.112	0.067	0.127	0.448 *	−0.268	0.071	0.058	0.160	0.171	0.010	0.233	−0.037	0.188	1

** Correlation is significant at the 0.01 level, * Correlation is significant at the 0.05 level. N = 21, Chla = Chlorophyll a (mg g^−1^ FW), Chlb = Chlorophyll b (mg g^−1^ FW), Chla + b = Chlorophyll a + b (mg g^−1^ FW), Car = Carotenoids (mg g^−1^ FW), Vit C = Vitamin C (mg g^−1^ FW), AC = Antioxidant capacity (%), TPC = Total polyphenol content (mg GA 100 g^−1^ FW), TFC = Total flavonoid content (mg CAE 100 g^−1^ FW), ACNs = Anthocyanins (mg 100 g^−1^ FW), Nit = Nitrate (mg kg^−1^ FW), Hue = Hue angle, Ch = Chroma, AI= APCI index, Y = Yield (kg m^−2^).

**Table 3:** Mean comparison of leaf color profile, APCI index, and yield of basil microgreen cultivars and genotypes.

Cultivars and Genotypes	a*	L*	b*	Hue	Chroma	APCI Index	Yield (kg m^−2^)	APCI Index × Yield
	(−60/+60)	(0–100)	(−60/+60)	(0–360)°	√(a^2^ + b^2^)			
Persian Ablagh	−22.41 ^g^	51.55 ^d^	28.30 ^f^	179.10 ^b^	36.10 ^g^	70.30 ^a^	3.34 ^ab^	234.80 ^a^
Dark Opal	−13.24 ^e^	42.94 ^j^	21.05 ^h^	178.99 ^b^	24.87 ^i^	46.05 ^g^	1.42 ^hi^	65.39 ^fg^
Amethyst Improved	6.41 ^b^	30.85 ^l^	13.00 ^j^	181.11 ^a^	14.49 ^l^	55.59 ^d^	2.32 ^cdefgh^	128.97 ^cd^
Red Rubin	8.82 ^a^	39.74 ^k^	16.58 ^i^	181.08 ^a^	18.78 ^k^	55.57 ^d^	3.16 ^abcd^	175.60 ^bc^
Italian large leaf	−26.34 ^j^	50.02 ^def^	37.42 ^bc^	179.04 ^b^	45.76 ^abc^	56.33 ^d^	1.98 ^ghi^	111.53 ^e^
Thyrsiflora	−25.05 ^hij^	57.79 ^b^	36.35 ^bc^	179.03 ^b^	44.15 ^c^	33.88 ^l^	3.60 ^a^	121.97 ^d^
Cinnamon	−26.02 ^j^	55.03 ^c^	36.92 ^bc^	179.04 ^b^	45.17 ^bc^	36.71 ^jk^	3.01 ^abcde^	110.50 ^e^
Persian green basil	−24.11 ^ghi^	49.34 ^efg^	30.77 ^e^	179.09 ^b^	39.09 ^e^	34.91	1.79 ^ghi^	62.49 ^g^
Persian purple basil	5.46 ^b^	45.47 ^i^	25.11 ^g^	181.36 ^a^	25.70 ^i^	38.18 ^ij^	3.03 ^abcde^	115.69 ^de^
Basilico Rosso	2.66 ^c^	39.28 ^k^	14.37 ^j^	181.39 ^a^	14.61 ^l^	45.64 ^g^	3.39 ^a^	154.72 ^c^
Kapoor	−25.55 ^hij^	48.15 ^gh^	35.90 ^c^	179.05 ^b^	44.06 ^c^	63.71 ^b^	3.24 ^abc^	206.42 ^ab^
lettuce leaf basil	−24.54 ^hij^	50.82 ^de^	33.51 ^d^	179.06 ^b^	41.53 ^d^	51.15 ^f^	2.19 ^efghi^	112.02 ^e^
Classic Italiano	−25.95 ^ij^	60.91 ^a^	39.25 ^a^	179.01 ^b^	47.05 ^a^	42.43 ^h^	3.54 ^a^	150.20 ^c^
Genovese	−26.26 ^j^	55.00 ^c^	37.84 ^ab^	179.04 ^b^	46.06 ^ab^	42.44 ^h^	3.77 ^a^	160.00 ^c^
Lemon	−24.00 ^gh^	51.45 ^d^	34.06 ^d^	179.04 ^b^	41.67 ^d^	31.05 ^m^	1.35 ^i^	41.92 ^h^
Mobarake	−26.37 ^j^	53.51 ^c^	37.05 ^bc^	179.05 ^b^	45.48 ^abc^	58.15 ^d^	2.05 ^fghi^	119.21 ^d^
Clove	−24.04 ^gh^	48.59 ^fg^	29.98 ^ef^	179.11 ^b^	38.43 ^ef^	39.78 ^i^	2.42 ^bcdefg^	96.27 ^ef^
Minimum	−22.68 ^g^	46.60 ^hi^	29.18 ^ef^	179.09 ^b^	36.96 ^fg^	33.57 ^l^	2.45 ^bcdefg^	82.25 ^f^
Blue Spice	−25.96 ^ij^	54.21 ^c^	37.26 ^bc^	179.04 ^b^	45.41 ^abc^	35.25 ^kl^	1.95 ^ghi^	68.74 ^fg^
Violetto	−8.99 ^d^	42.47 ^j^	21.41 ^h^	178.83 ^b^	23.22 ^j^	60.97 ^c^	2.98 ^abcdef^	181.69 ^b^
Hoary	−20.25 ^f^	45.52 ^i^	25.23 ^g^	179.11 ^b^	32.35 ^h^	50.64 ^f^	2.29 ^defgh^	115.97 ^de^
Species	***	***	***	***	***	***	***	***

*** Significant at *p* ≤ 0.001. Numbers with common letters in each column do not have a significant difference according to the Duncan test (*p* = 0.05).

### 2.8. Leaf Color Profile

The results showed that one of the most important factors affecting the color and lightness of microgreen basil leaves can be the difference in genotypes in terms of the color composition of the leaves, such as chlorophylls and anthocyanin, which directly affects the choice of consumers. The highest and lowest values of color component a* were measured in the Red Rubin cultivar (+8.82) and Mobarake genotype (−26.37), respectively. Also, the highest and lowest values of color component b^*^ were measured in the classic Italian cultivar (+39.25) and Amethyst cultivar (+13.00), respectively. Similarly, the highest and lowest L^*^ values were measured in the classic Italian cultivar (60.91) and Amethyst cultivar (30.85), respectively. The highest and lowest Hue angles (color angle) were measured in the Basilico Rosso cultivar (181.39°) and Violeto cultivar (178.83°), respectively. The highest and lowest Chroma (color intensity) were measured in the Classic Italian cultivar (47.05) and Amethyst cultivar (14.49), respectively.

### 2.9. Antioxidant Potential Composite Index (APCI)

**Figure 1:**
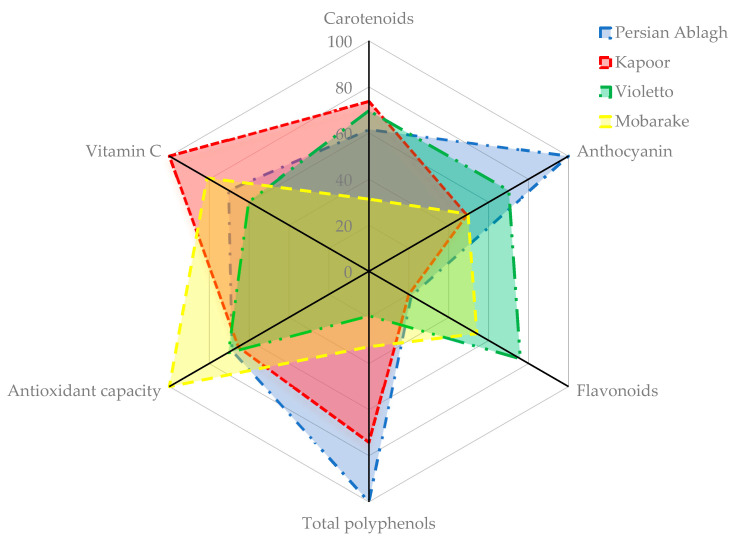
APCI index profiles of the four best basil microgreens.

**Figure 2:**
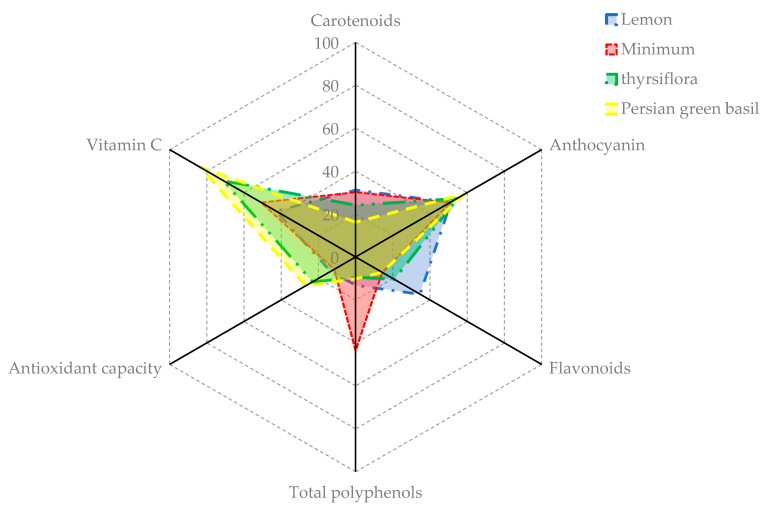
APCI index profiles of the four worst basil microgreens.

The antioxidant potency composite index (APCI) was utilized to rank the basil microgreens, as shown in Table 3. According to the APCI index score, the highest APCI index was related to the Persian Ablagh basil genotype (70.30), which was about 50% higher than the average APCI index of all cultivars (46.77). In the present study, it was found the Persian Ablagh basil genotype was a plant species with a high concentration of phenolic and anthocyanin compounds (Figure 1) and the lowest APCI related to the minimum basil variety (Figure 2).

### 2.10. Yield

The yield of basil microgreens (Table 3) ranged from 1.35 kg m^−2^ (lemon basil) to 3.77 kg m^−2^ (Genovese).

### 2.11. APCI Index × Yield (AY Index)

Based on the results of Table 3, the highest and lowest levels of antioxidant potential composite index based on the yield were measured in Persian Ablagh (234.80) and lemon basil (41.92).

### 2.12. Cluster Analysis

**Figure 3:**
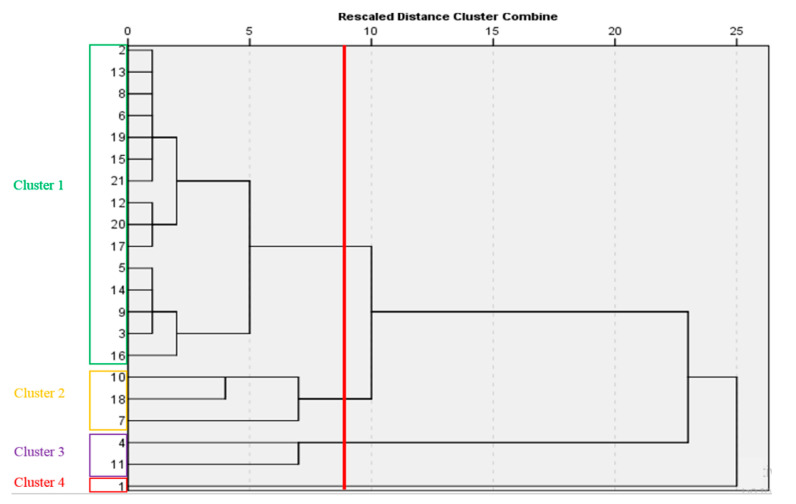
Dendrogram clustering graph of the 21 basil cultivars in this study based on phytochemical compounds and yield. 1-Persian Ablagh, 2-Dark Opal, 3-Amethyst Improved, 4-Red Rubin, 5-Italian large leaf, 6-thyrsiflora, 7-Cinnamon, 8-Persian green basil, 9-Persian purple basil, 10-Basilico Rosso, 11-Kapoor, 12-Lettuce leaf basil, 13-Classic Italian, 14-Genovese, 15-Lemon basil, 16-Mobarake, 17-Clove, 18-Minimum, 19-Blue Spice, 20-Violeto, 21-Hoary.

Basil cultivars and genotypes were clustered by using hierarchical cluster analysis based on their phytochemical characteristics and yield, and the results are displayed in Figure 3. The largest cluster was related to cluster 1, which included fifteen cultivars (Dark Opal, Classic Italian, Persian green basil, thyrsiflora, Blue Spice, Lemon basil, Hoary, Lettuce leaf basil, Violeto, Clove, Italian large leaf, Genovese, Persian purple basil, Amethyst Improved and Mobarake). This cluster had the lowest amount of antioxidant capacity, TPC, and yield. Cluster 2 included three cultivars (Basilico Rosso, Minimum, and Cinnamon) with the lowest carotenoids, anthocyanin, vitamin C, and APCI index. Cluster 3 included two cultivars (Red Rubin and Kapoor), which had the highest vitamin C, TPC, carotenoids, antioxidant capacity, and APCI index. In cluster 4, there was only one cultivar, Persian Ablagh, and it was specified by the high APCI index (no significant difference with cluster 3) among the clusters as well as the highest levels of anthocyanin and yield.

### 2.13. Principle Component Analysis (PCA)

**Figure 4:**
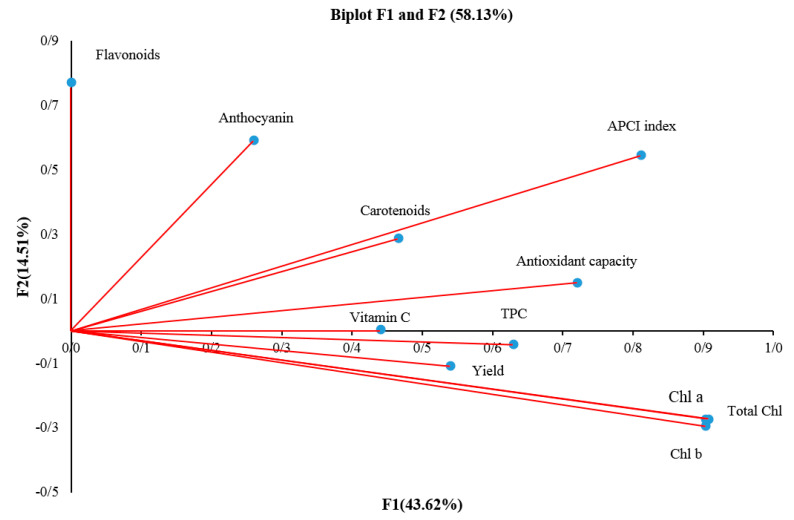
Biplot graph of PCA analysis for biochemical compounds and yield.

Examining the results of the eigenvalues and eigenvectors of the principle components analysis showed that factors 1–4 had a change rate of 78.12%, which is shown in Figure 4 and Table 4 and Table 5. As for the eigenvectors (Table 5), it was found that the chlorophyll a, chlorophyll b, total chlorophyll, antioxidant capacity, TPC, APCI index, and yield for F1 (43.62%) and the flavonoids, anthocyanin and APCI index for F2 (14.51%) should be appraised. In the biplot analysis graph (Figure 4), the F1 axis (43.62%) had the most impact related to chlorophylls and APCI index, and the F2 axis (14.51%)had the most important traits, including flavonoid and anthocyanin contents.

## 3. Discussion

The chlorophyll contents of basil microgreens were in agreement with Bulgari et al. [11], who assessed chlorophyll content in basil, Swiss chard, and rocket microgreens, demonstrating that these products typically have chlorophyll a (0.61–0.74 mg g^−1^ FW), chlorophyll b (0.19–0.26 mg g^−1^ FW) and total chlorophyll (0.77–1.00 mg g^−1^ FW) in the mentioned ranges. Previous studies have described various aspects of the antioxidant capacity of chlorophylls, the main conclusion being that chlorophylls are converted to their respective pheophorbide and pheophytin and are absorbed at the same rate as carotenoids, and in this way, pheophorbide a absorbed in the surface area of the intestines by the mechanism of the SR-BI mediated selective, which is an acceptable carrier, is transported to the intestine [12]. Considering the correlation of total chlorophyll with TPC and antioxidant capacity, the high chlorophyll content, especially chlorophyll a, in basil may be responsible for its high content of TPC and antioxidant capacity. In addition, it was stated in past research that the light received by chlorophyll changes cell metabolism and the synthesis of secondary metabolites such as TPC and antioxidant compounds [13].

In general, about 1100 carotenoids have been identified, which are divided into two groups: carotenes and xanthophylls [14]. In thylakoid membranes as photosynthetic tissues, carotenoids and chlorophylls act in absorbing light and performing photoprotective functions by inactivating free radicals, singlet O_2_, the triplet state of molecular structures of porphyrin (chlorophylls and protoporphyrin), and other reactive oxygen species [15]. Therefore, we expect carotenoids to act as antioxidants in any organism independent of its photosynthetic status. Kim et al. [16] pointed out that carotenoids are affected by pre-harvest factors such as the genetics of different varieties and species and the stage of the plants at harvesting time. The results showed that basil microgreens contain high amounts of β-carotene, the most carotenoids in leafy vegetables, and that the amount of carotenoids is highly dependent on the genotype; these results are in agreement with reports of results of past research [17,18].

The results were similar to and higher than the amount reported by Ghoora et al. [19] in studies of ten microgreens which reported that the content of vitamin C was in the range of 41.6–139.8 mg 100 g^−1^ FW. In an experiment reported by Kyriacou et al. [20], changes in ascorbic acid concentration in microgreens were a strong genotype-dependent effect. 

In past research, it has been stated that the production of vitamin C may be subject to different mechanisms, one of which is the L-galactose pathway using mannose or galactose (as a precursor) [21], as a response to oxidative stress; however, the general mechanism of vitamin C production is still not fully known. Vitamin C is a required cofactor for numerous enzymatic activities and a strong antioxidant, and, unlike most vitamins that can be synthesized within the body, it is not synthesized by humans, so it is necessary to get it from exogenous sources such as fruits and leafy vegetables [22]. Evidence from biochemical, clinical, and epidemiological studies suggests the consumption of 95 mg day^−1^ on average of vitamin C to optimally reduce the risk of chronic diseases such as hypertension, coronary heart disease, and stroke [23]. The basil microgreens we studied showed higher vitamin values than products known for their high ascorbic content, for example, oranges and peppers, with amounts of 49.4 and 50.3 mg 100 g^−1^ FW vitamin C, respectively [24]. Similarly, vitamin C values obtained for basil microgreens were higher compared with strawberries [24]. These values are also in agreement with the values detected in other microgreen species, such as carrots, onions, spinach, and radish, which are comparable and higher [19]. Therefore, the consumption of leafy vegetables such as basil microgreens, unlike cooked foods, helps to stabilize vitamin C in the plant tissue; thus, basil microgreens such as Kapoor, Hoary, and Persian green basil can be a natural and sustainable source of vitamin C for consumers.

Dhaka et al. [25], in an experiment, evaluated six microgreen samples for antioxidant capacity and reported that microgreens have values in the range of 72–87%. Comparing the results of this research with previous results shows that basil microgreens were as diverse as different plant genera in terms of antioxidant capacity. In recent years, due to the possible toxicity and adverse effects of synthetic antioxidants, there has been great interest in identifying natural sources of antioxidants, especially those of plant origin [26,27]. In the biosynthetic pathways of phytochemical products, plants produce secondary metabolites such as flavonoids, essential oils, alkaloids, lignans, terpenes, terpenoids, tocopherols, phenolic acids, phenolics, peptides, and organic acids, which the most important compounds with antioxidant ability, including vitamin C, tocopherols, carotenoids and flavonoids, but generally, phenolic compounds are the main sources of plant antioxidant compounds that exist in all plant parts [28,29]. Among the cultivars and genotypes of basil, the Mobarake genotype, Persian Ablagh genotype, and Violeto cultivar had the highest antioxidant capacity and can be recommended to consumers.

Xiao et al. [30] reported that in 30 varieties of commercial varieties of microgreens, the polyphenol compounds have a range of 88.60–811.00 mg GA 100 g^−1^ FW. The amount of TPC in most basil microgreens in this experiment is in the previously reported range, but the Persian Ablagh genotype had the highest amount of TPC that was detected in this experiment. Basil microgreens had a wide range of changes in terms of TPC, which could be caused by different internal factors (genetic) [19,25]. All TPCs are produced through phenylpropanoid metabolism in the shikimate pathway, which starts with the reaction of L-phenylalanine or tyrosine and conversion to trans-cinnamic acid, which causes the production of other phenols along the pathway, including cinnamic acid, benzoic acid, flavonoids, proanthocyanidins, stilbenes, coumarins, and lignins, which are mainly found in fruits and vegetables [31,32]. The TPC of plants is dependent on the balance between their synthesis and oxidation, and as antioxidants, TPC can be oxidized to quinone under oxidative stress. It is well known that after consuming foods rich in polyphenols, the absorption of polyphenols from the intestine has a direct relationship with the increase in the antioxidant capacity of blood plasma. The rate of absorption and speed limitation of polyphenols in the intestine is related to their chemical properties and structure. They are hydrolyzed by intestinal enzymes such as lactase and β-glycosidases or by colon microforms and then absorbed [33]. Therefore, based on the results of this experiment, basil microgreens can be considered excellent sources of these phytochemicals. The average requirement of phenolic acid in the human diet is reported to be about 200 mg day^−1^, depending on dietary preferences and habits [34]. Therefore, according to the results and the difference between basil microgreens in terms of phenolic compounds, consuming approximately 15–150 mg day^−1^ of basil microgreens would meet people’s daily needs.

The total flavonoid content was within the range reported in two basil microgreen varieties (2–10 mg CAE 100 g^−1^ FW) [7]. Another critical component of microgreens is the content of flavonoids, which are polyphenols with widely differing structures, which are basically classified as flavones, flavonols, flavanones, flavanonols, flavanols, isoflavones, anthocyanins, and chalcones [35,36]. They have in common an immunomodulatory impact on the human immune system, and their differing structures may be related to a few properties, including antioxidant, antibacterial, anticancer, antimutagenic, and anti-inflammatory effects. In this research, a significant difference was observed between cultivars and genotypes of basil microgreens in terms of flavonoid content, which indicates that this relationship is dependent on the species [37]. The shikimate pathway, which links flavonoid biosynthesis and nitrogen metabolism in plants, catalyzes carbohydrates from glycolysis and pentose phosphate pathways to synthesize aromatic amino acids (phenylalanine, tyrosine, and tryptophan) [38]. Lillo et al. [39] showed that photosynthetic carbon supply from the shikimate pathway is important for flavonoid synthesis, where flavonoids are synthesized from phenylalanine. As a result, flavonoid biosynthesis is regulated by nitrogen availability through photosynthetic carbon allocation among different biochemical pathways, which confirms the correlation between nitrates and flavonoids.

Anthocyanins may be considered defensive flavonoids whose synthesis is associated with additional cellular energy costs and cellular force energy that can delay plant metabolisms [40]. Some of the analyzed pigments, especially anthocyanins, whose content is high in red basil cultivars and genotypes, also apparently contribute to the total antioxidant capacity [41]. In addition, several authors have claimed in their research that the red pigment indicates the total content of anthocyanin and TPC [42]. These results explain the correlation found in basil microgreens between anthocyanin and APCI index, which are the main parts of antioxidant and polyphenolic compounds. The production and activity of anthocyanin is influenced by genetic factors and environmental factors such as light, so its concentration is expected to be different between different species, although its production does not follow the same pattern. Therefore, apart from the clear gap in the relative abundance of anthocyanins between green and purple microgreens, the amount of anthocyanin depends greatly on the genotype, and the selection of the highest amount of anthocyanin based on visual quality should be defined for a specific genotype. Generally, according to the anthocyanin color index and its relationship with basil microgreen antioxidant compounds, it can be concluded that red-colored plant species have more antioxidant potential, as observed in Dark Opal, Red Rubin, and Persian Ablagh basil.

In this experiment, it was proven that plant species may influence the nitrate content. In previous research, nitrate content in microgreens was reported between 310 and 1111 mg kg^−1^ FW, which was very dependant on the species [40]. Having unique photosynthetic and metabolic characteristics can lead to the accumulation of these compounds in plants, and such variation between plants can be related to the abilities of different plant species in nitrate accumulation [43]. Generally, microgreens are considered low nitrate accumulators among plant food products. However, in the research of Teng et al. [44], relatively high concentrations of nitrates have been observed in some microgreen species. Nitrate absorption, transfer, and accumulation in plants is controlled by several internal factors (genetic diversity, coordinated gene expression, enzyme activity, and ontogenetic stages) and external factors (nitrogen form, concentration, time of application, light intensity, quality and photoperiod, air temperature, and CO_2_ concentration), which affects the sensory and phytochemical characteristics of fresh vegetables [45]. As stated by Colla et al. [46], the use of genotypes with low nitrate accumulation can be considered to reduce nitrate content in plants. However, the levels of nitrates determined in basil microgreens were lower than the maximum levels of nitrates in leafy vegetables (2000–7000 mg kg^−1^ FW) reported in the European Commission Regulation (EC) [47]. Considering the difference between basil microgreens in nitrate content (Table 1), taking into account the maximum nitrate levels, our results show that different varieties and genotypes of basil microgreens can be produced in the floating system without the negative effect of nitrate content. Microgreens, as a healthy food, is necessary to reduce the consumption of food products with high nitrate content and increase the consumption of essential nutrients [48]. 

The coloration of microgreens constitutes one of the indirect measurements of quality, especially the organoleptic quality and, to some extent, phytochemical content, which influence the choice of microgreens by, and their economic value to, consumers, since visual quality is an essential component of current regulatory factors that set specific product quality standards for horticultural products [20]. Chlorophyll, carotenoids, and anthocyanins are responsible for the specific coloration of microgreens [49]. The increase in leaf redness can be due to the increase in anthocyanin content because a significant positive correlation between plant redness and anthocyanin content has been reported [50]. One of the most important functions of these pigments is visual appeal for consumers; in addition, they can be very useful for the immune system and human health due to their antioxidant properties [17]. The results of the research are consistent with the results of Ciriello et al. [51], who stated that b * values show a cultivar dependent effect, and Classic Italian records the highest b* (+21.62) compared with other cultivars. From the present study, it is concluded that the genotype is the main factor determining the color characteristics of basil microgreen leaves, especially brightness or darkness, overall color intensity, and color, which is according to the results of Kyriacou et al. [20].

In the present study, it was found the Persian Ablagh basil genotype was a plant species with a high concentration of phenolic and anthocyanin compounds (Figure 1). The previously conducted research has firmly corroborated that temperature and other abiotic factors stimulate the production of phenolic compounds within plants, thereby conferring resistance against these challenges [52]. In this pathway, key biosynthetic enzymes were activated, and essential genes involved in the phenylpropanoid pathway, including PAL and CHS, were upregulated, leading to an increase in TPC biosynthesis [53]. In response to temperature stress (both high and low), plants produce increased amounts of TPC compounds, including anthocyanins, flavonoids, flavonols, and phenolic acids, to safeguard their cells [54]. The origin of the collection of this genotype (Tabriz city) is a cold and dry climate (Supplementary Table S1), and considering that the basil plant is a product of the warm season, one of the factors that increase the antioxidant potential of this genotype is the eco-physiological conditions of that region. The production of these secondary metabolites may have increased the resistance of this plant to abiotic stresses. The lowest APCI index was found in the minimum basil variety (Figure 2), because it has less content of carotenoids, vitamin C, and anthocyanin compared with other cultivars and genotypes, and in the previous research by Žlabur et al. [55], it was stated that the minimum basil cultivar has fewer chlorophylls, vitamin C, and carotenoid than other cultivars, which is consistent with the results of this research.

The yields of basil microgreens (Table 3) ranged from 1.35 kg m^−2^ (lemon basil) to 3.77 kg m^−2^ (Genovese) and was more than that reported by Bulgari et al. [11], who used a shorter growing period and picked microgreens at the first leaf stage. The result of yield was consistent with the Kyriacou et al. [45] report, which determined that 10 microgreen species yielded more than 3 kg m^−2^, but the authors had opted for a lengthier growth duration akin to this study and harvested the microgreens at the second leaf stage. Žlabur et al., [55] reported the yield value of basil microgreens in the floating system was between 1.82–4.01 kg m^−2^. For the produce microgreens, large amounts of seeds (approximately 10,000 to 40,000 seeds m^−2^) are used to compensate for the low biomass of a single plant due to harvesting in the early stages of growth. However, the low yield remains a limiting factor for the microgreen industry [56]. The performance we observed in basil was within the range previously reported for this vegetable in the form of microgreen production. The optimal harvesting stage for microgreens should be the time that ensures high yield and, at the same, time high sensory quality and high nutritional value. Therefore, one of the goals of microgreen production, considering their lower performance compared with mature plants, can be the selection of cultivars and genotypes that have both good performance and a high antioxidant capacity index. In this research, the Genovese basil cultivar has the highest yield and can be used by producers to produce more fresh biomass, but if the production of basil microgreens to produce secondary metabolites with antioxidant properties, along with the performance, is suitable, among the genotype cultivars in the studied cultivars, the Persian Ablagh genotype, Violeto cultivar, and Kapoor cultivar can be more suitable options according to the APCI index and fresh biomass.

## 4. Materials and Methods

### 4.1. Plant Materials and Cultivation System

**Figure 5:**
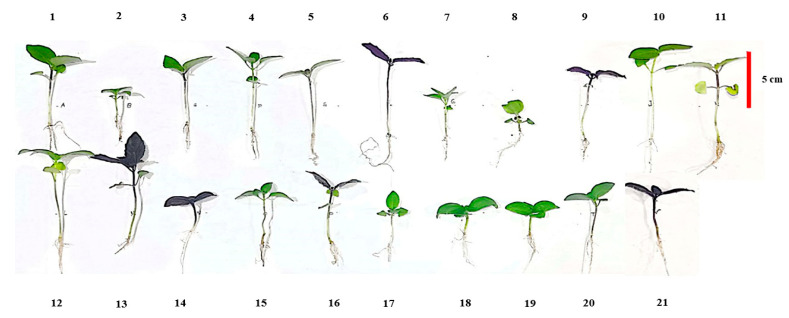
Images of basil microgreens in harvest step. 1-Blue Spice, 2-Minimum, 3-Cinnamon, 4-Italian large leaf, 5-Lettuce leaf basil, 6-Basilico Rosso, 7-Kapoor, 8-Hoary, 9-Violeto, 10-Mobarake, 11-Persian Ablagh, 12-thyrsiflora, 13-Red Rubin, 14-Amethyst, 15-Lemon basil, 16-Persian purple basil, 17-Clove, 18-Genovese, 19-Classic Italian, 20-Persian green basil, 21-Dark Opal.

In this study, 17 basil cultivars (Genovese, thyrsiflora, Violeto, Classic Italian, Basilico Rosso, Kapoor, Red Rubin, Cinnamon, Clove, Amethyst, Hoary, Lettuce leaf basil, Minimum, Dark Opal, Italian large leaf, Blue Spice and Lemon) from the company Imported seeds (seed bank of southern Iran and Emanuele Larosa Seeds from Puglia, Italy) and 4 genotypes of Iranian basil (Persian green basil, Persian purple basil, Persian Ablagh basil, and Mobarake) were obtained from local farmers (Table S1 and Figure 5). Basil seeds were grown in the floating system using an LED lamp with a combination of blue and red light (1:1) with 16 h of photoperiod and light intensity of 300±15 μmol photon m^−2^ s^−1^ in a completely randomized design (CRD) with 3 replications. In the floating system, seeds were cultivated in trays with 105 cells and a seed density of 48.5 g m^−2^ in a combination of coco-peat and perlite (*v*/*v*: 50%), and, for faster germination, the seed trays were kept in the dark for 48 h at a temperature of 27 °C and humidity of 60%. After seed germination, the day and night temperatures were adjusted to 24/22 ± 1 °C and the humidity was set at 65/75% ± 5% during the growth period, and for feeding of the microgreens, one-fourth modified Hoagland′s nutrient solution (nitrate 2 mM, sulfur 0.25 mM, phosphorus 0.20 mM, potassium 0.62 mM, calcium 0.75 mM, magnesium 0.17 mM, ammonium 0.25 mM, iron 20 μM, manganese 9 μM, copper 0.3 μM, zinc 1.6 μM, boron 20 μM, and molybdenum 0.3 μM), with electrical conductivity (EC) and pH of 0.5 ± 0.1 dS m^−1^ and 6 ± 0.2, respectively, were used [57]. In the floating system, microgreen seedling trays were placed in another tray with a volume of 12 L and a surface of 2 cm of nutrient solution, and to guarantee uniformity in light and humidity on the tray surface, rotation of trays every day was done.

### 4.2. Chlorophyll and Carotenoid Content

The measurement of photosynthetic pigment was done according to the Arnon method [58], with modifications for using a microplate reader (INNO, LTEK, Gyeonggi-do, Korea). Briefly, 30 mg of fresh leaves of basil were extracted using 300 μL of 80% acetone. After 72 h in the dark, the samples were mixed for 3 min, and 200 μL of the extract was placed in each microplate cell. The absorption was recorded using the above microplate reader at wavelengths of 663, 645, and 470 nm. Finally, the amounts of chlorophyll a, chlorophyll b, total chlorophyll, and carotenoids were obtained in terms of mg g^−1^ fresh weight using the Equations (1)–(4):

Chlorophyll a = (((12.7 × A663) − (2.69 × A645))/W) × V(1)

Chlorophyll b = (((22.9 × A645) − (4.68 × A663))/W) × V(2)

Total chlorophyll= (((20.08 A645) + (8.02 A663))/W) × V(3)

Carotenoids = (1000(A470) − 1.8 (chl a) − 58.2 (chl b))/198(4)

W = Sample weight (g), V = Sample volume (mL).(5)

### 4.3. Vitamin C

Vitamin C was measured according to the method described by OCHOA-VELASCO et al. [59]. Briefly, 0.3 g of fresh leaves of basil was mixed with 1 mL of 1% metaphosphoric acid solution. Then the mixture was centrifuged at 900 g for 15 min. The supernatant containing vitamin C was used for the color change reaction of the sodium salt of 6, 2-dichloroindophenol (DCIP) in the microplate. To perform this reaction, 70 μL of the extract was mixed with 70 μL of 30 ppm DCIP solution and the mixture stood at room temperature for 1 min. Finally, absorbance was measured at a wavelength of 515 nm by using a microplate reader. The content of vitamin C in the samples was determined using a calibration curve and was expressed in terms of mg AA g^−1^ FW (y = 629.42x − 5.3205, R² = 0.99).

### 4.4. Antioxidant Capacity, Polyphenols, Flavonoids, and Anthocyanin

The plant extract was prepared by extracting 0.5 g of fresh plant leaves with 5 mL of 80% methanol. After keeping the extract in the dark for 24 h at 4 °C, the extract was centrifuged at 3000 rpm for 15 min and the supernatant was collected to evaluate the content of biochemical compounds.

For measuring the total antioxidant capacity, a 126.8 μM solution of DPPH was prepared in methanol [60]. Finally, 20 μL of plant leaf extract, along with 290 μL of DPPH solution, were transferred to the micro-tube, and the absorbance at 515 nm was measured using a microplate reader after incubation for 16 min. The percentage of DPPH inhibition was calculated using the equation:

Radical scavenging activity DPPH % = {(Abs of control-Abs of sample)/(Abs of control)} × 100 

In order to measure polyphenols, first, 20 μL of sample extract was mixed with 20 μL of 10% (w/v) Folin–Ciocalteu reagent, and finally, 160 μL of 1M Na_2_CO_3_ was added to the solution [61]. After incubation for 20 min in the dark, absorbance was measured at 765 nm using a microplate reader. The total phenolic compounds in mg g^−1^ FW was calculated from the calibration curve of the gallic acid (0.01–0.1 mg mL^−1^, R^2^ = 0.99).

In order to measure flavonoids, 20 μL of the extracted sample was added to a mixture of 85 μL of distilled water and 5 μL of 5% NaNO_2_. After 6 min of reaction, 10 μL of 10% AlCl_3_.6H_2_O was added to the mixture. After 5 min of reaction, 35 μL of 1M NaOH and 20 μL of distilled water were added to the mixture, and the absorbance was measured using the microplate reader at a wavelength of 520 nm, and the results were expressed as mg of (+)-catechin (CAE) hydrate g^−1^ FW (y= 875.44x-55.709, R² = 0.99) [62].

In order to measure the amount of anthocyanin in the extract, two buffers with different pH were prepared: potassium chloride pH = 1 (0.025 M), pH adjusted with HCl using a pH meter, and sodium acetate pH = 4.5 (0.4 M). In the next step, 40 μL of basil extract and 160 μL of buffer were added to the microplates well, and absorbance was at 520 and 700 nm wavelengths using the above microplate reader device after 20–50 min [63].

### 4.5. Nitrate Concentration

Nitrate concentration was determined using the microplate spectrophotometer method modified by Hachiya and Okamoto [64]. A quantity of 50 mg of frozen microgreen powder was transferred to a tube and mixed with 500 μL of deionized water and then was held in a boiling water bath for 20 min. After cooling to room temperature, the sample was centrifuged at 3000 rpm for 10 min. A quantity of 10 μL of the extract was mixed with 40 μL of salicylic acid in 0.05% (*w/v*) sulfuric acid and then incubated at room temperature for 20 min. After that, 1 mL of 8% NaOH (*w/v*) was added to each sample. Absorbance was measured at 410 nm by using a microplate reader, and the amount of nitrate was calculated using the nitrate standard curve in mg kg^−1^ of FW (y = 5149x − 60.696, R^2^ = 0.99).

### 4.6. Leaf Color Profile

The color profile of the second microgreen basil leaf was measured immediately after harvest with a colorimeter (Chroma Meter CR-400; Konica Minolta Sensing, Inc., Kyoto, Japan) using a*, lightness (L*) and b* coordinates. Then, Hue (angle index) and Chroma (color saturation measure) were calculated using the following Equations (5) and (6) [65]:

Hue angle = tan^−1^(a*/b*) (6)

Chroma = √ (a^2^ + b^2^) (7)

### 4.7. Antioxidant Potential Composite Index (APCI)

To quantitatively evaluate the antioxidant potential of basil microgreens, the combined antioxidant potential composite index (APCI) was used [19]. Equal weights were assigned to all antioxidant compounds. First, individual APCI was calculated by assigning a score of 100 to the highest value in each assay and subsequently calculating the score for all other samples as a percentage of the highest score. The APCI index was the average of six antioxidant activity indices, including antioxidant capacity, polyphenols, vitamin C, flavonoids, anthocyanin, and carotenoids.

### 4.8. Yield of Basil Microgreen

The yield of basil microgreens on the 30th day after sowing the seeds was measured using a digital scale and yield was reported in kg m^−2^.

### 4.9. APCI Index × Yield (AY Index)

In order to measure the APCI index based on yield, the amount of APCI index was multiplied by the yield at harvesting time.

### 4.10. Statistical Analysis

The data were analyzed using IBM SPSS statistical software version 22, and Duncan′s multiple range test was used to compare the means. One-way analysis of variance (ANOVA) was used to test the statistical significance effect on studied strains. To determine if there was a linear correlation between studied strains, a bivariate Pearson correlation analysis was conducted. By using of hierarchical cluster analysis, the different types of basil were categorized to determine the relatedness of their biochemical composition and yield. A dendrogram plot was employed to visualize the final results of cluster analysis, which employed between-groups linkage and Euclidian distance measurement intervals. Principle component analysis (PCA) was carried out using IBM SPSS statistical software to examine relationships between basil genotypes’ biochemical compounds.

## 5. Conclusions

Different chemotypes of microgreen basil cultivars based on the APCI index and biochemical compounds were revealed through hierarchical cluster analysis. According to the results, different basil genotypes had different plant phytochemicals according to their collection areas. Considering that the environmental stresses (such as high temperature, low temperature, salinity, etc.) are different in the origins of the seeds, basil cultivars and genotypes produce one or more types of secondary metabolites more than other genotypes to adapt to those stresses. This caused the Kapoor cultivar to produce more chlorophylls, carotenoids, vitamin C, TPC and AC; the Violetta cultivar to produce a higher amount of carotenoids, antioxidants, and flavonoids; and the Persian Ablagh genotype to produce more carotenoids, TPC and ACNs than other basil genotypes. The AY index determined based on antioxidant potential and fresh biomass could easily distinguish between genotypes and identify and recommend the best ones for further studies. Therefore, it can be concluded that the genetic characteristics, i.e., basil cultivars and genotypes, have an effect on the antioxidant compounds of basil microgreens. According to the results, it is possible to recommend the Persian Ablagh basil genotype and Kapoor and Violeto cultivars for the production of microgreens based on the APCI index, AY index, and their fresh biomass to nutrition experts for selecting and recommending vegetables rich in nutrients.

## Figures and Tables

**Table 1:** Mean comparison of biochemical and antioxidant traits of basil microgreen cultivars and genotypes.

Cultivars and Genotypes	Chl a	Chl b	Chl a + b	Carotenoids	Vitamin C	Antioxidant Capacity (%)	Polyphenols	Flavonoids	Anthocyanin	Nitrate
	(mg g^−1^ FW)	(mg g^−1^ FW)	(mg g^−1^ FW)	(mg g^−1^ FW)	(mg g^−1^ FW)		(mg GA 100 g^−1^ FW)	(mg CAE g^−1^ FW)	(mg 100 g^−1^ FW(	(mg kg^−1^ FW)
Persian Ablagh	0.65 ^bcd^	0.17 ^bc^	0.83 ^bc^	0.20 ^abcd^	1.69 ^bcde^	60.28 ^b^	1463.79 ^a^	1.68 ^g^	26.19 ^a^	533.29 ^defg^
Dark Opal	0.41 ^hij^	0.10 ^hij^	0.51 ^gh^	0.06 ^d^	1.56 ^bcdef^	35.14 ^cd^	146.64 ^g^	3.94 ^cde^	24.74 ^a^	155.98 ^i^
Amethyst Improved	0.27 ^k^	0.08 ^j^	0.35 ^i^	0.15 ^bcd^	1.77 ^bcd^	8.26 ^g^	486.17 ^de^	7.95 ^a^	18.78 ^bcde^	551.31 ^cdef^
Red Rubin	0.66 b^cd^	0.16 ^cd^	0.82 ^bcd^	0.17 ^abcd^	1.71 ^bcd^	23.56 ^cdefg^	1210.21 ^b^	1.72 ^g^	21.05 ^b^	484.37 ^efgh^
Italian large leaf	0.48 ^efghi^	0.13 ^efg^	0.62 ^efg^	0.28 ^ab^	1.13 ^def^	31.42 ^cd^	432.52 ^de^	6.23 ^b^	16.58 ^cdefgh^	740.96 ^ab^
Thyrsiflora	0.59 ^cde^	0.15 ^cde^	0.74 ^cde^	0.08 ^cd^	1.71 ^bcd^	20.24 ^defg^	135.88 ^g^	1.64 ^g^	14.35 ^fgh^	147.11 ^i^
Cinnamon	0.50 ^efgh^	0.13 ^efg^	0.63 ^efg^	0.07 ^cd^	1.06 ^ef^	25.06 ^cdef^	848.10 ^c^	1.21 ^ghi^	14.18 ^fgh^	607.09 ^bcde^
Persian green basil	0.34 ^jk^	0.10 ^hij^	0.44 ^hi^	0.05 ^d^	2.00 ^abc^	23.52 ^cdefg^	148.76 ^g^	1.14 ^ghi^	15.32 ^efgh^	95.62 ^i^
Persian purple basil	0.43 ^fghij^	0.11 ^ghi^	0.53 ^fgh^	0.05 ^d^	1.19 ^def^	13.08 ^efg^	404.46 ^def^	3.58 ^e^	19.84 ^bcd^	142.82 ^i^
Basilico Rosso	0.41 ^hij^	0.11 ^ghi^	0.52 ^gh^	0.08 ^cd^	1.45 ^cdef^	54.49 ^b^	723.69 ^c^	0.63 ^i^	18.30 ^bcdef^	358.22 ^gh^
Kapoor	0.93 ^a^	0.24 ^a^	1.18 ^a^	0.24 ^abc^	2.40 ^a^	57.49 ^b^	1085.53 ^b^	1.58 ^g^	12.83 ^h^	721.22 ^abc^
lettuce leaf basil	0.73 ^b^	0.19 ^b^	0.92 ^b^	0.20 ^abcd^	1.27 ^def^	37.89 ^c^	253.05 ^efg^	4.53 ^c^	20.15 ^bc^	866.54 ^a^
Classic Italiano	0.40 ^hij^	0.12 ^fgh^	0.52 ^gh^	0.21 ^abcd^	1.46 ^cdef^	31.34 ^cd^	148.23 ^g^	1.46 ^g^	17.21 ^bcdefg^	126.51 ^i^
Genovese	0.36 ^ijk^	0.09 ^ij^	0.45 ^hi^	0.14 ^bcd^	1.29 ^def^	19.60 ^defg^	445.00 ^de^	3.79 ^de^	15.23 ^efgh^	651.71 ^bcde^
Lemon	0.40 ^hij^	0.10 ^hij^	0.50 ^gh^	0.10 ^bcd^	1.03 ^f^	11.78 ^efg^	191.29 ^fg^	2.74 ^f^	13.38 ^gh^	327.33 ^h^
Mobarake	0.55 ^def^	0.14 ^def^	0.68 ^def^	0.10 ^bcd^	1.95 ^abc^	86.76 ^a^	479.11 ^de^	4.30 ^cd^	13.01 ^gh^	675.46 ^bcd^
Clove	0.54 ^defg^	0.14 ^def^	0.68 ^def^	0.24 ^abc^	1.25 ^def^	20.57 ^defg^	291.53 ^efg^	0.77 ^hi^	15.71 ^defgh^	130.81 ^i^
Minimum	0.39 ^hijk^	0.11 ^ghi^	0.50 ^gh^	0.10 ^bcd^	1.22 ^def^	10.08 ^fg^	638.72 ^cd^	1.13 ^ghi^	13.32 ^gh^	403.70 ^fgh^
Blue Spice	0.42 ^ghij^	0.12 ^fgh^	0.54 ^fgh^	0.08 ^cd^	1.52 ^bcdef^	26.45 ^cde^	153.17 ^g^	2.36 ^f^	14.08 ^gh^	601.94 ^bcde^
Violetto	0.69 ^bc^	0.17 ^bc^	0.86 ^bc^	0.23 ^abcd^	1.45 ^cdef^	61.86 ^b^	281.64 ^efg^	6.03 ^b^	18.36 ^bcdef^	744.39 ^ab^
Hoary	0.31 ^jk^	0.09 ^ij^	0.40 ^hi^	0.33 ^a^	2.11 ^ab^	22.31 ^cdefg^	180.53 ^fg^	1.38 ^gh^	15.93 ^defgh^	108.40 ^i^
Species	***	***	***	***	***	***	***	***	***	***

*** Significant at *p* ≤ 0.001. Numbers with common letters in each column do not have a significant difference according to the Duncan test (*p* = 0.05).

**Table 4:** Eigenvalues of the principal components for biochemical compounds of basil microgreen genotypes.

Component	Initial Eigenvalues	Extraction Sums of Squared Loadings	
	Total	% of Variance	Cumulative %
1	4.80	43.62	43.62
2	1.60	14.51	58.13
3	1.17	10.66	68.79
4	1.03	9.33	78.12

**Table 5:** Eigenvectors of the principal components for biochemical contents of basil microgreen genotypes.

	F1	F2	F3	F4
Chlorophyll *a*	0.90	−0.27	−0.07	−0.28
Chlorophyll *b*	0.90	−0.29	−0.11	−0.24
Total chlorophyll	0.90	−0.27	−0.07	−0.27
Carotenoids	0.46	0.28	−0.46	0.04
Vitamin C	0.44	0.00	−0.30	0.75
Antioxidant capacity	0.72	0.15	−0.06	0.16
Total Polyphenols	0.63	−0.04	0.49	0.16
Total flavonoid content	0.00	0.77	−0.28	−0.39
Anthocyanin	0.26	0.59	0.61	−0.04
APCI index	0.81	0.54	−0.03	0.15
Yield	0.54	−0.10	0.36	0.08

The red numbers have a significant strong effect in each factor (F).

## Data Availability

Data will be made available on request.

## Supplementary Materials

The following are available online at https://www.mdpi.com/article/10.3390/plants12142652/s1, Table S1: Average annual temperature of the origin of microgreen basil genotypes..
