# Supplementary material for: Biochemical Compounds, Antioxidant Capacity, Leaf Color Profile and Yield of Basil (Ocimum sp.) Microgreens in Floating System
Source: Plants (Basel). 2023 Jul 14;12(14):2652. doi: 10.3390/plants12142652 (PMC10386441; doi:10.3390/plants12142652)
Supplement: Supplementary file 1 [file plants-12-02652-s001.zip › plants-2498839-supplementary.pdf]

Table S1. Average annual temperature of the origin of microgreen basil genotypes.

Cultivar/genotype	Origins (City, Country)	AVG of annual temperature (°C)	Classification of climate (Köppen-Geiger)
Persian Ablagh	Tabriz, Iran	10.6	Csa
Dark Opal	Mansfield, United States	10.3	Dfa
Amethyst Improved	Genoa, Italy	12.5	Cfb
Red Rubin	Copenhagen, Denmark	8.9	Cfb
Italian large leaf	Milan, Italy	15.8	Csa
thyrsiflora	Bangkok, Thailand	27.7	Aw
Cinnamon	Mexico, Mexico	15.9	Cwb
Persian green basil	Ahvaz, Iran	26.9	BSh
Persian purple basil	Ahvaz, Iran	26.9	BSh
Basilico Rosso	Andria, Italy	17.0	Csa
Kapoor	Kapoor, India	25.3	Cwa
lettuce leaf basil	Tokyo, Japan	15.2	Cfa
Classic Italiano	Genoa, Italy	12.5	Cfb
Genovese	Genoa, Italy	12.5	Cfb
Lemon	New Delhi, India	24.6	BSh
Mobarake	Isfahan, Iran	16.7	BWk
Clove	Antananarivo, Madagascar	17.9	Aw
Minimum	Rimini, Italy	15.1	Cfa
Blue Spice	Semo Smrzice, Czech Republic	8.6	Cfb
Violetto	Andria, Italy	17.0	Csa
Hoary	New Delhi, India	24.6	BSh

All data are taken from https://en.climate-data.org. Cwa = Monsoon-influenced humid subtropical climate, BSh = semiarid climate found in the low latitudes, Csa = Hot-summer Mediterranean climate, Dfa = Hot-summer humid continental climate, Cfb = Temperate oceanic climate or subtropical highland climate, Aw: tropical savanna climate with dry-winter characteristics, Cwb = Subtropical highland climate or Monsoon-influenced temperate oceanic climate, Cfa = Humid subtropical climate, BWk = Cold desert climate.
